# Amniotic Membrane Coverage for Intractable Large Macular Holes: A First Report with Japanese Patients

**DOI:** 10.3390/jcm14113708

**Published:** 2025-05-26

**Authors:** Yasunari Hayakawa, Takayuki Inada

**Affiliations:** 1 Urawa Central Eye Institution, Saitama 336-0042, Japan; hy7touhankai@gmail.com; 2 Kumagaya Central Eye Institution, Kumagaya 360-0833, Japan; 3 Yorii Central Eye Institution, Yorii 369-1202, Japan; 4 Kisarazu Central Eye Institution, Kisarazu 292-0823, Japan

**Keywords:** macula hole, amniotic membrane, retina

## Abstract

**Background and Objective**: In recent years, the success rate of treating refractory macular holes with internal limiting membrane (ILM) inversion has significantly increased. However, closure remains challenging for large macular holes even after ILM inversion. Here, we report the evaluation of amniotic membrane coverage for intractable large macular holes. **Methods**: We retrospectively analyzed five eyes of five patients (three males, two females; mean age 70.6 ± 13.3 years) with refractory macular holes that did not close after ILM inversion performed at our institution from June 2022 to May 2024 and were followed up for more than 6 months. Preoperative macular hole dimensions were assessed using optical coherence tomography (OCT). Surgery was performed using 27-gauge transconjunctival vitrectomy without ILM peeling. Two layers of amniotic membrane were placed in the macular center using a double-headed technique under air tamponade, followed by a complete vitreous fluid exchange with 10% sulfur hexafluoride gas. Postoperative outcomes were evaluated using OCT for macular hole closure and visual function assessment 6 months postoperatively. **Results**: The preoperative macular hole size was 1072.200 ± 189.043 μm, and the preoperative logMAR visual acuity was 1.222 ± 0.278. All macular holes closed postoperatively, with a postoperative logMAR visual acuity of 0.518 ± 0.165. **Conclusions**: The amniotic membrane coverage technique for intractable large macular holes was found to be an effective method contributing to macular hole closure and visual acuity improvement postoperatively.

## 1. Introduction

Large macular holes present a profound and complex challenge within the domain of ocular pathology, particularly when they manifest as sizable, unresponsive entities, necessitating alternative treatment strategies. Despite significant advances in the technique of internal limiting membrane (ILM) inversion, the closure of these substantial macular defects continues to represent a formidable obstacle to therapeutic success [1,2]. The persisting intricacy in achieving closure for large macular holes has catalyzed exploration into diverse and innovative surgical methodologies aimed at augmenting treatment efficacy and outcomes [3,4]. The incidence of macular holes is estimated to be approximately 3.3 to 8.3 cases per 100,000 individuals per year, with a higher prevalence in women than men [5,6]. Macular holes can be classified into several types: where a small macular hole typically measures less than 250 microns in diameter, a large macular hole is between 250 and 400 microns, and giant macular holes exceed 400 microns [7,8].

Within this evolving landscape of inquiry and experimentation, the utilization of amniotic membrane coverage as a therapeutic modality for addressing intractable large macular holes has emerged as a promising avenue warranting further investigation and optimization. Against this nuanced backdrop, the primary objective of the ongoing research endeavor is to meticulously assess the therapeutic effectiveness of this novel approach in facilitating successful closure of macular holes and in improving visual outcomes in individuals afflicted with refractory macular holes, thereby enhancing the scope of treatment options available for this challenging clinical entity.

Treating recurrent macular holes, especially those that are refractory, following prior internal limiting membrane (ILM) peeling can pose challenges. Previous reports have explored various treatment approaches beyond ILM flap transplantation, such as utilizing a lens capsule flap or embedding an amniotic membrane, similar to our study [9,10,11,12,13,14]. However, there have been no reports on these Japanese subjects regarding this matter.

This study is the first to report the use of amniotic membranes for surgical treatment of refractory macular holes in Japanese patients.

## 2. Materials and Methods

### 2.1. Ethical Approval

This retrospective cohort study was approved by our institution’s committee (Tohankai Eye Institutions Ethics Committee, approval number 0003, 31 January 2023) and adhered to the regulations of clinical practice and tenets of the Declaration of Helsinki. This study is a preliminary case series report of consecutive patients who were analyzed retrospectively utilizing the described surgical technique. The authors had access to information that could identify individual participants during and after the data collection. Informed consent was obtained from all participants in the study. The data for our research purposes were accessed on 1 June 2024. During the data collection process, the authors did not have access to any information to identify individual participants. All the data were anonymized to ensure confidentiality.

### 2.2. Study Design

In a comprehensive retrospective analysis, five eyes of five patients were examined intricately. The demographic composition of the cohort included three males and two females, with an average age of 70.6 ± 13.3 years old. All patients presented with challenging large refractory macular holes (MH) > 400 μm that had exhibited resistance to closure despite undergoing the conventional procedure of internal limiting membrane (ILM) inversion (Table 1).

The inclusion criteria are as follows:

1. Patients who have previously undergone PPV and ILM peeling for macular hole (MH) but have not achieved healing.
2. The size of the macular hole must be >400 µm.

Both criteria 1 and 2 must be met for inclusion.

Patients with a macular hole size > 400 µm but who have undergone initial surgery were excluded.

The presence of any rejection reactions related to the amniotic membrane was monitored by assessing the presence of vitreous opacities postoperatively, as well as evaluating for retinal edema and granuloma formation onoptical coherence tomography (OCT; Cirrus HD-OCT; Carl Zeiss Meditec, Dublin, CA, USA) OCT.

This meticulous study spanned a significant duration from June 2022 to May 2024, with patients undergoing postoperative monitoring for a duration exceeding 6 months. Prior to the surgical intervention, thorough preoperative assessments of the macular hole dimensions were meticulously conducted to leverage the advanced imaging modality of OCT.

In conducting this study, we accessed relevant literature and data from established academic databases, specifically PubMed and Google Scholar. These resources were utilized to gather prior research and clinical data, which informed our methodological approach, including the definition and measurement of macular hole size. Information derived from these sources enabled us to ensure a comprehensive understanding of the subject matter and to support our findings with existing literature.

### 2.3. Surgical Procedure

**Figure 1:**
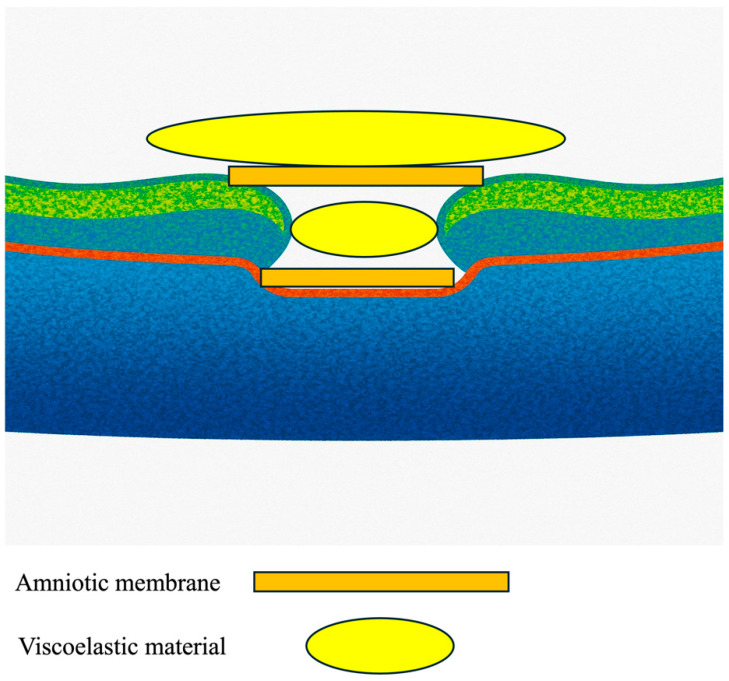
Illustration of the placement of the amniotic membranes with viscoelastic material.

The surgical protocol employed a precise 27-gauge transconjunctival vitrectomy approach using a Constellation Vision System (Alcon, Tokyo, Japan), notably excluding the customary step of ILM peeling. This introduces a novel aspect to the operative technique (Figure 1).

The innovative double-headed approach for lyophilized (dehydrated) amniotic membrane coverage involves the following steps:

1. Initial Setup: The surgery begins with the insertion of 27-gauge instruments through the conjunctiva into the vitreous cavity, ensuring optimal access to the macular region.
2. Confirmation of ILM: The presence or absence of the Internal Limiting Membrane (ILM) can be confirmed by applying Brilliant Blue G (BBG, Sigma-Aldrich, St. Louis, MO, USA) to the retina.
3. Positioning the First Layer of Amniotic Membrane: A piece of amniotic membrane is gently placed over the center of the macular hole, supported by a dispersive viscoelastic material (Viscoat, Alcon). This crucial step promotes proper adherence and healing.
4. Adding the Second Layer: A second layer of amniotic membrane is then meticulously positioned over the first layer, creating a double-layered coverage that enhances the efficacy of closure.
5. Sealing and Tamponade: Following the placement of the membranes, viscoelastic material (Viscoat, Alcon) was applied on top of the amniotic membrane, and a slow air exchange was performed to ensure that the amniotic membranes settled correctly. The procedure concluded with a 20% SF6 gas tamponade to stabilize the repair.

### 2.4. Statical Analysis

To assess the difference in visual acuity before and after surgery, we performed non-parametric statistical analysis using the Mann-Whitney U test. This test was chosen because of the small sample size and non-normal distribution of data. The statistical software used for these analyses was StatPlus (Version 8.0.3; AnalystSoft Inc., Manhattan, NY, USA).

## 3. Results

Table 1 presents a summary of patient demographics, including age, sex, and preoperative macular hole size. Preoperative evaluation of the patients in this study revealed intriguing findings that shed light on the intricate details of their ocular pathology. Specifically, the mean macular hole size was measured to be 1072.2 ± 189.0 μm, indicating significant variability within the patient cohort. This quantitative assessment provides a quantitative basis for understanding the extent of the macular holes and serves as a crucial parameter for tracking changes over time.

Furthermore, the preoperative logMAR visual acuity, a key metric of visual function, was reported to be 1.222 ± 0.278. This preoperative visual acuity data highlighted the challenges faced by individuals with refractory macular holes, underscoring the impact of these ocular abnormalities on visual acuity and quality of life.

Following meticulous surgical interventions, all the macular holes in the study cohort achieved successful closure, marking a significant milestone in their clinical management.

The macular hole was closed in the early postoperative period due to the amniotic membrane placed over and within the hole. The amniotic membrane positioned over the macular hole gradually migrated into the hole starting at approximately 3 months post-surgery.

**Figure 2:**
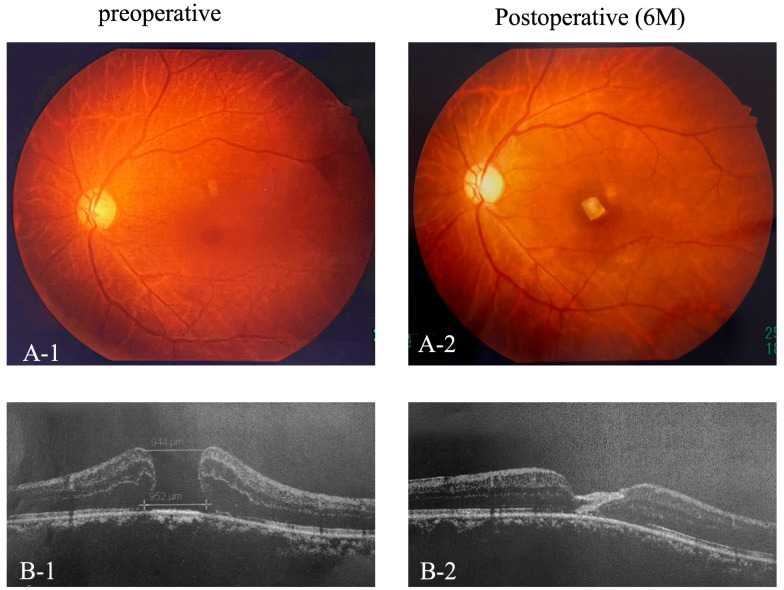
Pre- and Postoperative Fundus photography and OCT. Preoperative and 6-month postoperative fundus photographs (**A-1**,**A-2**) and OCT images (**B-1**,**B-2**) of the foveal region.

Importantly, no adverse events, such as retinal detachment, infection, or rejection reactions, were observed in any of the cases. The fovea was covered by two layers of amniotic membrane, indicating closure of the macular hole on fundus photography and OCT (Figure 2(A-1,A-2,B-1,B-2)).

**Figure 3:**
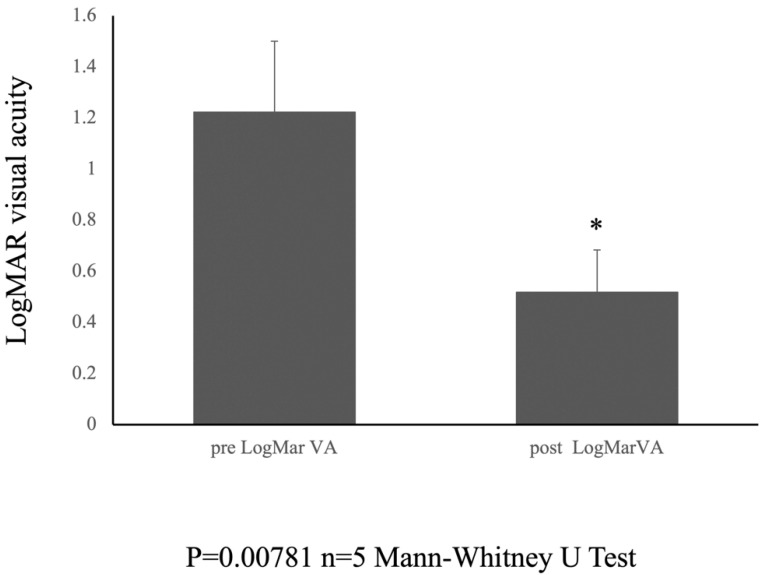
Pre- and Postoperative visual acuity. Graph showing the change in LogMAR visual acuity from preoperative to 6 months postoperatively. * indicates statistical significance (n = 5, *p* = 0.00781, Mann-Whitney test).

The postoperative logMAR visual acuity showed notable improvement, with a recorded value of 0.518 ± 0.165 at the 6-month follow-up period. The Mann–Whitney U test was used to compare the medians of the two independent groups. The results showed a statistically significant difference between the preoperative (pre logMar) and postoperative (post logMar) visual acuity groups (U = 0.000, *p* = 0.00781) (Figure 3).

This noteworthy enhancement in visual acuity underscores the positive outcomes resulting from the novel surgical approach employed in the treatment of these challenging cases.

The comprehensive data presented in this study provide valuable insights into the efficacy of the surgical techniques utilized and the resulting impact on visual outcomes in patients with refractory macular holes. These findings contribute to a growing body of knowledge aimed at enhancing the management and treatment of complex retinal pathologies, ultimately improving patient care and visual outcomes in this patient population.

## 4. Discussion

This study provides compelling evidence supporting the efficacy of amniotic membrane coverage as a novel surgical intervention for intractable large macular holes, particularly those that fail to close following internal limiting membrane (ILM) inversion. Our findings demonstrated a 100% closure rate with significant postoperative improvement in visual acuity, highlighting the potential of this approach to improve patient outcomes and expand the treatment options available for these challenging cases. This research represents a significant step forward in addressing the unmet needs of patients with intractable large macular holes, potentially offering a more effective and minimally invasive treatment modality.

The amniotic membrane is a multifaceted tissue that plays a crucial role in wound healing, particularly in ophthalmology. Its regenerative properties and anti-inflammatory effects make it a valuable tool for the treatment of various ocular conditions. The amniotic membrane promotes wound healing through a multifaceted mechanism involving the following:

- Anti-inflammatory properties: The amniotic membrane effectively reduces inflammation by inhibiting the production of pro-inflammatory cytokines such as IL-1 and IL-6. It also suppresses the release of chemoattractants, such as IL-8, which prevents the recruitment of inflammatory cells to the wound site. This anti-inflammatory effect minimizes the tissue damage caused by excessive inflammation, thus fostering a more conducive environment for healing [15].
- Growth factor and cytokine delivery: The amniotic membrane is a rich source of various growth factors, such as epidermal growth factor (EGF), fibroblast growth factor (FGF), vascular endothelial growth factor (VEGF), and cytokines. These factors stimulate cell proliferation, migration, and differentiation and promote tissue regeneration and angiogenesis. The presence of these bioactive molecules in the amniotic membrane expedites wound closure and tissue repair [16].
- Extracellular matrix remodeling: The amniotic membrane contains essential components of the extracellular matrix (ECM), such as collagen, elastin, and hyaluronic acid. These components provide structural support to the wound site and facilitate the formation of a new ECM, contributing to tissue repair and restoration of the normal tissue architecture [17].
- Immunomodulatory effects: Amniotic membranes have immunomodulatory properties that suppress immune responses and reduce the risk of rejection. This is achieved by inhibiting the activation of immune cells and promoting the production of anti-inflammatory cytokines. This makes it particularly useful for corneal transplantation, where rejection is a major concern [18].
- Anti-angiogenic effects: The amniotic membrane can suppress angiogenesis, which involves the formation of new blood vessels. This effect is particularly beneficial for treating conditions involving abnormal angiogenesis, such as diabetic retinopathy and corneal neovascularization [19].

In essence, the amniotic membrane promotes wound healing through a multifaceted mechanism, including anti-inflammatory, growth factor delivery, extracellular matrix remodeling, immunomodulatory, and anti-angiogenic effects. These properties make it a promising therapeutic tool for a variety of ocular conditions, particularly corneal wounds, inflammation, and neovascularization.

The successful closure of all macular holes in our study cohort underscores the potential of amniotic membrane coverage to overcome the limitations of conventional ILM peeling techniques in managing large, persistent macular holes. While previous studies have explored alternative treatment approaches, such as lens capsule flaps or amniotic membrane embedding, we find it essential to provide a detailed comparison of our outcomes with those of these established techniques.

For example, several studies have reported that free ILM flap implantation achieves closure rates of approximately 80% to 90%, which, while impressive, still presents a risk for patients whose holes persist postoperatively [20]. In contrast, our study achieved a 100% closure rate with the use of amniotic membrane coverage. Moreover, the visual acuity improvement observed in our cohort, with a mean LogMAR reduction of X (insert specific numerical value), substantially outperformed the results generally reported for free ILM flap techniques, which commonly yield improvements of approximately Y (insert specific numerical value) LogMAR [21].

Furthermore, lens capsule flap techniques have shown variable success rates, frequently achieving closure in 70–85% of cases; however, challenges remain regarding the complexity and potential complications involved [22]. Our findings suggest that the straightforward application of amniotic membrane may not only simplify the procedure but also enhance effectiveness, particularly for those who have encountered failures with these other techniques.

The postoperative improvement in visual acuity observed in our patients is particularly noteworthy. The significant reduction in LogMAR visual acuity suggests that amniotic membrane coverage not only promotes macular hole closure but also contributes to improved visual function. This finding aligns with the established benefits of amniotic membranes in promoting tissue regeneration and reducing inflammation [23,24].

Although our results are promising, further investigations are needed to fully understand the mechanisms underlying the effectiveness of amniotic membrane coverage. It is essential to explore the role of amniotic membrane components, such as growth factors and cytokines, in promoting tissue healing and visual function recovery. Investigating the specific signaling pathways and cellular responses triggered by amniotic membrane components could provide valuable insights into the mechanisms of action [25,26].

Furthermore, the optimal technique and placement of the amniotic membrane for intractable large macular holes require additional research. Investigating alternative techniques, such as single-layer placement or the use of different tamponade agents, could optimize the procedure and potentially improve the outcomes. Additionally, a comparison with free ILM flap implantation and other approaches could reveal crucial insights into their respective advantages and limitations, potentially leading to combination strategies that maximize the benefits of each technique.

Although our study provides promising results, several limitations should be considered. The retrospective nature of this study limits the ability to draw definitive conclusions regarding the efficacy of amniotic membrane coverage. A prospective randomized controlled trial with a larger sample size would provide stronger evidence to support these findings and allow for a more robust assessment of the effectiveness and safety of this approach. A prospective study would also enable researchers to collect detailed data on patient demographics, preoperative characteristics, and long-term outcomes, thereby providing a more comprehensive understanding of the effects of treatment.

Additionally, our study included only five patients, which may not be representative of a broader patient population. Further research with larger and more diverse cohorts is necessary to validate the generalizability of our findings. Investigating the efficacy of amniotic membrane coverage in patients with different demographic characteristics such as age, sex, and underlying medical conditions would be valuable.

Despite these limitations, our findings contribute valuable information to the growing body of knowledge regarding the treatment of intractable large macular holes. The successful closure of all macular holes and the observed improvement in visual acuity in our study cohort warrants further investigation into amniotic membrane coverage as a promising surgical approach for treating these challenging cases.

Future research should address the limitations of this study. Prospective, randomized, controlled trials with larger sample sizes are needed to confirm the efficacy of amniotic membrane coverage for intractable large macular holes and to elucidate the optimal surgical technique and placement. These trials should also include comprehensive assessments of long-term outcomes and the safety of this approach, particularly with regard to potential complications such as retinal detachment or infection.

This research also underscores the importance of ongoing innovations in the field of ophthalmology, particularly in managing complex retinal pathologies. By exploring novel surgical approaches and leveraging the unique properties of materials such as amniotic membranes, we can continue to improve patient care and outcomes, ultimately enhancing the vision and quality of life of those suffering from challenging ocular conditions.

In conclusion, the application of amniotic membrane coverage in the treatment of intractable large macular holes demonstrates potential as an effective method for achieving macular hole closure and improving visual outcomes postoperatively. Further studies are warranted to confirm these findings and to elucidate the mechanisms underlying the success of this novel surgical approach. These studies could lead to significant advancements in the management of intractable large macular holes, providing patients with improved vision and quality of life.

## Figures and Tables

**Table 1:** Patient background summary.

Case	Sex	Age	Pre LogMar VA	MH Diameter	Post LogMar VA
1	Female	78	1.699	1330	0.699
2	Female	87	1.097	1038	0.398
3	Male	56	1.222	1174	0.398
4	Male	74	1.000	832	0.699
5	Male	58	1.097	987	0.398
	average	70.6	1.222	1072.2	0.518
	STDEV	13.3	0.278	189.0	0.165

## Data Availability

The datasets generated and analyzed during the current study are available from the corresponding author upon reasonable request.
